# Simultaneous estimation of the temporal and spatial extent of animal migration using step lengths and turning angles

**DOI:** 10.1186/s40462-023-00444-8

**Published:** 2024-01-08

**Authors:** Peter R. Thompson, Peter D. Harrington, Conor D. Mallory, Subhash R. Lele, Erin M. Bayne, Andrew E. Derocher, Mark A. Edwards, Mitch Campbell, Mark A. Lewis

**Affiliations:** 1 Department of Biological Sciences, University of Alberta, Edmonton, AB Canada; 2 Department of Mathematics, University of British Columbia, Vancouver, BC Canada; 3 Department of Mathematical and Statistical Sciences, University of Alberta, Edmonton, AB Canada; 4 Inuit Tapiriit Kanatami, Ottawa, ON Canada; 5 Office of the Chief Scientist, Environment and Protected Areas, Government of Alberta, Edmonton, AB Canada; 6 Department of Renewable Resources, University of Alberta, Edmonton, AB Canada; 7 Government of Nunavut, Iqaluit, NU Canada; 8 Department of Biology, University of Victoria, Victoria, BC Canada; 9 Department of Mathematics and Statistics, University of Victoria, Victoria, BC Canada

## Abstract

**Background:**

Animals of many different species, trophic levels, and life history strategies migrate, and the improvement of animal tracking technology allows ecologists to collect increasing amounts of detailed data on these movements. Understanding when animals migrate is important for managing their populations, but is still difficult despite modelling advancements.

**Methods:**

We designed a model that parametrically estimates the timing of migration from animal tracking data. Our model identifies the beginning and end of migratory movements as signaled by change-points in step length and turning angle distributions. To this end, we can also use the model to estimate how an animal’s movement changes when it begins migrating. In addition to a thorough simulation analysis, we tested our model on three datasets: migratory ferruginous hawks (*Buteo regalis*) in the Great Plains, barren-ground caribou (*Rangifer tarandus groenlandicus*) in northern Canada, and non-migratory brown bears (*Ursus arctos*) from the Canadian Arctic.

**Results:**

Our simulation analysis suggests that our model is most useful for datasets where an increase in movement speed or directional autocorrelation is clearly detectable. We estimated the beginning and end of migration in caribou and hawks to the nearest day, while confirming a lack of migratory behaviour in the brown bears. In addition to estimating when caribou and ferruginous hawks migrated, our model also identified differences in how they migrated; ferruginous hawks achieved efficient migrations by drastically increasing their movement rates while caribou migration was achieved through significant increases in directional persistence.

**Conclusions:**

Our approach is applicable to many animal movement studies and includes parameters that can facilitate comparison between different species or datasets. We hope that rigorous assessment of migration metrics will aid understanding of both how and why animals move.

## Introduction

Migration is one of the most widespread and important ecological processes within the animal kingdom [6, 19]. The process occurs in countless animal taxa and has evolved convergently many times [34, 75, 80]. Owing in part to this convergent evolution, migration is a diverse process, occurring across a wide variety of temporal and spatial scales [1, 9, 26, 47]. Understanding how and why animals migrate is important for advancing ecological theory but understanding where these animals are going, and when they get there, facilitates effective management of their populations [52, 66]. As the environment changes rapidly and unprecedentedly, the migratory patterns of many animals have changed in response, particularly with respect to their spatial and temporal extent [46, 91]. Modern tracking technology allows ecologists to collect animal location data at fine spatial and temporal resolutions, creating opportunities to answer more complex questions pertaining to migration [53]. This influx of data describes the spatial extents of many animal migrations in detail. The temporal extent of migration is needed for phenological studies but is more difficult to quantify.

Ecologists have designed many approaches to identify the beginning and end of an animal’s migration [12, 41, 87]. In some cases, the presence of ecological barriers along an animal’s migratory route make the onset of migration easy to classify without explicit modelling [81]. For example, López-López et al. [62] analyzed the migratory behavior of Eleonora’s falcons (*Falco eleonorae*) breeding on islands in the Mediterranean Sea, assuming their migrations began as soon as they left these islands. Statistical methods can estimate migration timings when simple heuristics cannot be defined as easily. There are many such methods but most of them fall within one of the following four categories [41]. Metric-based methods involve calculating secondary metrics from movement data (e.g., net squared displacement or NSD, the animal’s distance from its first recorded location). Classification methods typically involve manipulating or deriving the output of clustering algorithms, often with the assistance of machine learning, to assess significant changes in movement behaviour. Phenomenological methods treat location data as time series with some implicit periodicity or variability and attempt to identify the timescale of this pattern. Mechanistic methods attempt to model the underlying process that cases the migration (e.g., as a biased random walk or range shift). Some models may have qualities that match more than one of these categories, and all of them can produce the same output: estimated “change-points” that divide a path into migratory and non-migratory segments. These “path segmentation” analyses [21] can be applied at different time scales, from identifying area-restricted searching bouts in foraging animals [40, 97] to annual migration patterns [61, 63, 67, 99]. Many path segmentation approaches couple a movement metric (e.g., NSD) with a change-point algorithm that identifies changes in the distribution of this metric [21]. Change-point algorithms often use dynamic programming to efficiently and optimally segment time-series data, which relies on assumptions that each segment displays behaviour unrelated to the others [54, 57, 90]. This assumption may not hold for migrants, where we may expect separate migratory and/or non-migratory periods to exhibit similar patterns. Most (albeit, not all) path segmentation analyses also only use one metric to identify migration [21], potentially omitting information that could be useful in estimating the phenology of this complex process.

Identifying when animals migrate is difficult, and solving this problem has taken attention away from characterizing how animals migrate in the process. Dingle and Drake [19] provide two separate definitions for migration in individual animals: a persistent period of directionally autocorrelated (or straight) movement, and a period of movement ranging over an exceptionally large spatial extent. While these definitions are a broad generalization of the many diverse ways animals migrate, they suggest that characterizing the behavioral changes observed during migration can elucidate important qualities about an animal’s ecology. Step lengths, the Euclidean distance between two consecutive tracked locations, and turning angles, the angle made by the animal’s turn during three consecutive tracked locations, describe distance and direction, respectively. By accounting for the temporal difference between tracked locations they can be used to evaluate an animal’s speed and tortuosity. Both of these metrics are widely used in movement ecology [4, 32, 68, 92]. Both of the aforementioned definitions of migration imply movement between spatially disjoint regions [19], but we suggest that each definition is linked to step lengths and turning angles separately. The first definition of migration suggested by Dingle and Drake [19] relates to directional persistence, and could be quantified by a change in an animal’s turning angles, while the second definition relates to distance covered and could be quantified by a change in an animal’s step lengths. A path segmentation model that identifies simultaneous changes in two metrics (step lengths and turning angles) will allow ecologists to draw more biological context from migration data.

We designed a simple path segmentation model that identifies temporal changes in step lengths and turning angles calculated from discrete-time animal location data. We wanted to assess whether such a model could achieve this goal with as much accuracy, precision, and certainty as existing approaches. In situations when this is true, we believe that our approach is advantageous due to its biologically relevant, easy-to-interpret model parameters. Our multi-metric change-point (MMCP) model quantifies the hypothesis that migration can be quantified by an abrupt change in an animal’s observed movement rates for a sustained temporal interval. Unlike most path segmentation approaches, which focus on one all-encompassing movement metric, our model estimates distributions for step lengths and turning angles concurrently. We designed a likelihood-based method for identifying the optimal sequence of change-points (e.g., start and end of migration) and used a parametric bootstrapping algorithm to generate confidence intervals for the parameter estimates. We compared our model to a variety of other approaches (metric-based, classification, phenomenological, and mechanistic) using a thorough simulation analysis inspired by Gurarie et al. [41]. We also tested our model on three case studies: ferruginous hawks (*Buteo regalis*) in the Great Plains of central North America, and barren-ground caribou (*Rangifer tarandus groenlandicus*) and brown bears (*Ursus arctos*) in northern Canada.

## Methods

### The model

Our modelling approach is designed for discrete-time, continuous-space tracking data that samples an animal’s movement path throughout the landscape. The model identifies a given number of migratory periods from the data by analyzing the step lengths and turning angles generated by the discrete-time data. Many models that attempt to identify change-points (including hidden Markov models, which are widely applied across ecology) do not estimate a fixed number of change-points. Typically, when working with migratory animals, the number of desired change-points is known *a priori* and as a result, change-point algorithms without a fixed number of change-points need to be adjusted using post-hoc tools. The number of migrations included in our model, *c* can be any positive integer, but for many single-year tracking datasets, identifying $c = 2$ unique migratory periods will be most biologically useful. We define the model first for $c = 1$ for simplicity. The model relies on step lengths and turning angles, which can be calculated from consecutive tracked animal locations or steps. The animal’s step length at time *t*, which we denote $r_t$, is simply the Euclidean distance between its last two locations. Step lengths are an indicator of the distance an animal travels per time step, and turning angles indicate the directional persistence (or straightness) of movement [68]. The turning angle is the angle made between an animal’s current directional heading and its previous heading. Smaller turning angles (closer to 0 or 2$\pi$) indicate straighter movement paths. The interpretation of these metrics depends heavily on the temporal resolution at which they are calculated, so it is necessary that movement data be regularized to constant temporal intervals (i.e., every step lasts the same amount of time) before fitting the model. Our model assumes that step lengths and turning angles are independent, which may not always be true in animal movement data [49]. We also do not explicitly account for temporal autocorrelation between step lengths and turning angles.

Step lengths and turning angles are well-studied and can typically be explained effectively using known distributions, which we leverage for our model [3, 4]. We modelled animal step lengths with an exponential distribution at all stages of movement, but during the animal’s migratory stage, the parameter dictating the mean step length increases. We modelled turning angles with a von Mises distribution, where the angular concentration parameter increases during migration. Both of these distributions are not necessary for our model to work, and distributions with more parameters (e.g., gamma or Weibull for step lengths; [56]) or with a different shape (e.g., wrapped Cauchy for turning angles; [5]) could be substituted. When $c = 1$, we assume there exist two temporal parameters $t_1$ and $t_2$ ($0< t_1 < t_2$) that signal the start and end of migration, respectively. The likelihood function for any given point $\textbf{z}_t$ incorporates these conditions explicitly with model parameters $t_1$, $t_2$, $\rho _0$, $\rho _1$, $\kappa _0$, and $\kappa _1$. During the non-migratory period ($t < t_1$ or $t > t_2$) the animal’s step length distribution is parameterized by $\rho _0$ and the animal’s turning angle distribution by $\kappa _0$. The parameters $\rho _1$ and $\kappa _1$ represent the additional mean movement distance and angular concentration incurred during migration, respectively. We define the likelihood function for a single location $\textbf{z}_t$ as follows:

1 $$\begin{aligned}{} & {} I_{mig}(t) = {\left\{ \begin{array}{ll} 1 &{} t_1 < t \le t_2 \\ 0 &{} \text {otherwise}, \end{array}\right. } \end{aligned}$$

2 $$\begin{aligned}{} & {} L(\rho _0, \rho _1, \kappa _0, \kappa _1, t_1, t_2 | \textbf{z}_t) = \frac{\exp \left[ \left( -\rho _0 - I_{mig}(t)\rho _1\right) ^{-1}r_t + \left( \kappa _0 + I_{mig}(t)\kappa _1\right) \cos \phi _t\right] }{\left( \rho _0 + I_{mig}(t)\rho _1\right) \left( 2 \pi I_0(\kappa _0 + I_{mig}(t)\kappa _1)\right) }. \end{aligned}$$

Here, $I_0(\kappa )$ is the modified Bessel function of order 0. The likelihood for a dataset consisting of *T* steps is calculated as the product of Eq. 2 for all $\textbf{z}_t \in \{ \textbf{z}_1,..., \textbf{z}_T\}$. The ratio between the animal’s mean step length during and outside of migration approximates how much more quickly the animal moves when migrating. We denote this quantity $R = \frac{\rho _1 + \rho _0}{\rho _0}$.

If necessary, we can also expand the model to account for multiple migratory periods within one dataset. This would necessitate the introduction of additional parameters $t_3, t_4,..., t_{2c-1}, t_{2c}$ for a model with *c* distinct periods of migratory movement. If $c > 1$, $I_{mig}(t)$ would be 1 when $t_{2n-1} < t \le t_{2n}$ for any integer $n \in \{1, 2,..., c\}$. Unique step length and turning angle parameters ($\rho _2,..., \rho _c$ and/or $\kappa _2,..., \kappa _c$) for each migratory period could be biologically realistic for some species. For any positive integers *m* and *n*, where $m < n$, the *m*-migration model is nested within the *n*-migration model; this can be verified by setting all $\rho$ and $\kappa$ equal to each other and fixing all $t_i$ equal to each other for $i > 2m$.

### Parameter estimation

Optimizing the likelihood function (Eq. 2) is difficult because the function is not continuous or differentiable with respect to temporal parameters $t_1$ and $t_2$. However, when all $t_i$ parameters are fixed at a certain value, the function can be optimized easily. Since the step-length and turning-angle components of the likelihood function are independent the maximum likelihood estimates (MLE’s) for all $\rho _i$ and $\kappa _i$ values can be derived or approximated without the need for numerical optimization. The MLE for $\rho _0$ is simply the mean of all step lengths from any timesteps *t* satisfying $I_{mig}(t) = 0$. The MLE for $\kappa _0$ does not have a simple analytical expression, so we approximated it using the mle.vonmises function from the circular package of the R software [76], once again only considering turning angles calculated at timesteps where $I_{mig}(t) = 0$ [2]. To estimate $\rho _i$ and $\kappa _i$ parameters corresponding to migratory periods, we calculated the same MLEs for these periods and subtracted $\rho _0$ and $\kappa _0$, respectively. If either of these calculations produced negative numbers we replaced these estimates with 0, in line with the hypothesis that migration will be typified by faster and more directed movements. From these parameter estimates we calculated the optimal negative log-likelihood (NLL) value for any set of change-points (i.e., $t_i$ values) using Eq. 2.

We designed a change-point algorithm that, given the NLL for any combination of $t_i$ values, searches efficiently for the optimal change-points. Evaluating the NLL for every possible set of change-points, known as an “exhaustive search”, is an exact (i.e., guaranteed to find the optimal change-points) method but is extremely slow, especially as *c* increases. The algorithm employed by Lavielle [57], sometimes labeled as “optimal partitioning” [90], is exact and much more efficient, but only works for cost functions that can be calculated as a sum of independent components for each segment of the time-series. When we calculate the NLL, we assume that many segments have the same parameter values so these parameters (e.g., $\rho _0$ and $\kappa _0$) have clear biological interpretations. Since these existing approaches do not work for our problem, we designed a change-point algorithm that does not require this independence and also searches much more efficiently than an exhaustive search. The algorithm relies on similar $t_i$ values producing similar NLL values; in other words, the NLL for $(t_1, t_2)$ will not be very different from $(t_1 \pm g, t_2 \pm g)$, for some number *g*. If this is true, then identifying the lowest NLL along a (2*c*)-dimensional grid of resolution *g* should determine the general region where the optimal $t_i$ values lie. This optimal region can then be searched more thoroughly (i.e., over a smaller grid) to find the optimal value. We suggest that iteratively cutting the grid size in half (i.e., grids of size $g, \frac{g}{2}, \frac{g}{4},...$) until reaching the desirable minimum grid size $g_m$ is the most efficient way to search the parameter space. When *g* is not a power of 2, truncating decimal places such that all grid sizes remain integers may be desirable. Our algorithm is not exact but when the likelihood function is sufficiently smooth across $t_i$ values it will identify the global optimum.

We set the initial grid size *g* to 14 days and tried subsequently smaller grid sizes of 7 days, 3 days, and $g_m = 1$ day. The number of grids used and their respective resolution depends on the temporal extent of the data as well as the desired precision with which one hopes to estimate the $t_i$ parameters. For example, some animals may migrate in hours, which would necessitate using a minimum grid size reflecting that scale. Our choice of $g_m = 1$ day provides valuable inference for large-scale migrations and saves computational time that would be spent optimizing over finer grids. In other ecological systems, smaller minimum grid sizes may be necessary. For each grid, we identified the $t_i$ combinations that produced the five lowest NLL values and searched those optimal regions with the subsequent smaller grid; this accounts for parameter spaces with multiple local minima. We wrote the grid-search algorithm, which can loop over thousands of different $t_i$ values depending on the data, using the Rcpp R package, which seamlessly integrates R and C++ to increase computational efficiency [20].

We designed a parametric bootstrapping algorithm that estimates 95% confidence intervals (CI’s) for our model’s parameters. We cannot obtain CI’s using more standard methods (e.g., Wald-type estimations or likelihood profiles) because the likelihood function includes $I_{mig}(t)$, which is discontinuous and depends on the $t_i$ parameters. To generate CI’s for an individual migration, we simulated random paths with the same size and temporal extent as the true migratory path. The number of random paths necessary to generate consistent CI’s may vary depending on the dataset. These simulated paths were generated using the likelihood function and parameterized based on the MLE for each of the model parameters from the true path. We then fit the model to each of these paths independently and used the distribution of the parameter estimates from each random path to obtain CI’s (taking the 2.5% and 97.5% quantiles as lower and upper confidence bounds, respectively). The process of re-simulating data according to the estimated parameter values has been used to analyze time-series data for many purposes, including calculating CI’s [18, 55].

### Simulation analysis

We assessed our model’s ability to identify the temporal extent of migration from three separate simulated movement processes. We also used this simulation analysis to compare our model to other approaches used for the same task. The analysis here is heavily inspired by Gurarie et al. [41], as the paths we simulated for this analysis were generated from a nearly identical process to what was used there. Our simulation analysis included movement paths generated from three different models, each representing a potential mechanism for migration. All simulated paths lasted for 300 timesteps and contained a migration starting at timestep 100 and ending at timestep 200. Paths generated from the “speed switch” model were simulated from a continuous-time correlated velocity movement (CVM) model where the mean step length increased during migration. We also simulated paths from a CVM where speed remained constant but the timescale of autocorrelation, which influences movement directionality, increased during migration. This “timescale switch” model produced migratory paths while retaining a constant step length distribution throughout the process. The final set of paths were generated from a discrete biased correlated random walk, where the average speed and directionality of movement remained constant but the spatial location of bias changed at the beginning of migration. This “bias switch” model produced migratory paths without any explicit changes in the step length or turning angle distributions of the paths. We generated 50 random realizations of each process using the waddle R library designed by Gurarie et al. [41]. For the speed and timescale switch models, we simulated paths using the multiCVM function, and for the bias switch, we used the multiBCRW function. See Table 1 for the exact parameter values used for each set of paths.

**Table 1:** Parameter values used for each set of simulated paths

Parameter	Before migration ($t < 100$)	During migration ($100 \le t < 200$)	After migration ($t \ge 200)$
*Speed switch model*			
Mean speed, $\nu$	1	5	1
Autocorrelation timescale, $\tau$	2	2	2
*Timescale switch model*			
Mean speed, $\nu$	1	1	1
Autocorrelation timescale, $\tau$	2	20	2
*Bias switch model*			
Weibull step length parameters, $\alpha , \beta$	1, 1	1, 1	1, 1
Angular concentration parameter, $\kappa$	0.5	0.5	0.5
Attraction point, ($z_1$, $z_2$)	(0, 0)	(50, 0)	(50, 0)
Attraction strength, *A*	0.5	0.9	0.5

The functions provided by the waddle library produce complete paths with locations at evenly spaced timesteps, but real-life animal location data often come with timesteps in which locations are missing [33, 78]. We manually removed locations from each path with a probability of $\frac{1}{12}$ for each location. Location error is also a part of most animal tracking datasets, and even small errors can be magnified when calculating step lengths and turning angles [50, 51]. We jittered the x and y coordinates of every location in each path by a random number drawn from a Gaussian distribution with mean 0 and variance $\sigma _e$. We ran the full suite of simulation analyses for $\sigma _e = 0, 1,$ and 25.

We fit seven different models, including ours, to each of the 150 simulated paths and compared each model’s estimates for the beginning and end of migration (referred to as $\hat{t_1}$ and $\hat{t_2}$). While not exhaustive, owing to the large number of approaches used for this goal, our set of models includes a wide variety of mathematical approaches that leverage different metrics and quantities derived from animal movement data [21, 41]. We compared our model to the following six approaches: the non-linear regression model from Bunnefeld et al. [10] that assesses patterns in net squared displacement (NSD) over time; the first passage time (FPT) approach used by Le Corre et al. [58]; a Bayesian piecewise regression approach with NSD as the response variable [99]; the behavioral change point analysis designed by Gurarie et al. [40]; the mechanistic range shift analysis technique designed by Gurarie et al. [42]; and a Bayesian partitioning of Markov models (BPMM) approach applied to the time-series of log step lengths derived from the data [13, 37]. We also tracked how long each algorithm took to evaluate using the Sys.time R function. Absolute time values will not be generalizable across other machines, but since all our analyses were run on the same machine, these measurements allowed us to determine which algorithms were faster than others. All analyses were conducted using R 4.3.1 [76]. More details are provided in the Additional file 1.

### Case studies

#### Ferruginous hawks in the Great Plains

Ferruginous hawks are large, migratory raptors found in central Canada and the United States [84, 85]. The shortgrass prairies of southern Alberta, Canada represent the northern edge of this species’s breeding range, and birds breeding this far north make relatively long migrations to the southern Great Plains in the United States [96]. Adult ferruginous hawks were captured at nest sites during the breeding season, using either a dho-gazza net or a bal-chatri trap [94]. Captures were limited to nests in which the young had survived at least 10 days. Once captured, the birds were fitted with solar ARGOS/global positioning system (GPS) platform transmitter terminals and solar Groupe Special Mobile (GSM) tags. ARGOS tags recorded a location every 1 h and GSM tags recorded a location as frequently as every 1 min [94]. We rarefied each movement track to one location per day for consistency with our other case studies. Our dataset includes 50 individual hawks tagged on their breeding territories in southeastern Alberta and spans 10 years (2012–2021). The tags also provided estimates of dilution of precision (DOP) in the horizontal and vertical directions for every location. We removed any locations with a DOP over 5 in either the horizontal or vertical directions in preparation for our analysis [22].

We isolated each individual migration (fall or spring) temporally so we could fit our model with $c = 1$ to them separately. Each hawk was originally tagged on its breeding territory so we used the date at which the first location was received for each individual as the cut-off point between spring and fall. To define a cut-off between the end of fall migration and the beginning of spring migration (i.e., the birds’ arrival at the wintering grounds), we used the date at which the southernmost location was recorded in each year. This separation tactic may not apply well to other datasets (e.g., where migration is not clearly north-to-south). We removed any migrations that were missing a significant section of data, either spatially (any path containing a location that was further than 400 km away from the previous recorded location) or temporally (any path containing a 14-day period without any recorded locations). We bounded $t_1$ and $t_2$ such that $t_2 - t_1$ needed to be greater than 7 days, as anything shorter would represent a biologically unrealistic migration [96]. We estimated 95% confidence intervals for each individual migration using parametric bootstrapping. We simulated 100 random paths for each true migratory path. We ran the bootstrapping algorithm multiple times for the same migration and compared the intervals to ensure that this number of paths produced consistent CI’s.

Like many animal species, ferruginous hawks display complex migratory patterns including stopovers and pre-migratory dispersal [95, 96]. Stopover behaviour is defined as the interruption of migration over some temporal period [77] and is very diverse, just like migration itself [27, 82, 83]. Stopovers have many functions and differentiating long-term, foraging stopovers from shorter stopovers may be important in identifying critical habitat for migratory species [36]. During fall migration, many ferruginous hawks display long-term stopovers. Watson and Keren [96] consider these fall movements to be two separate migrations partitioned by the stopover. Ferruginous hawks also frequently embark on pre-migratory movements, where they disperse from their breeding or winter territory before returning to the same general area [95]. To evaluate whether our model could statistically identify stopovers and other complexities from the ferruginous hawk data, we compared our model fits with $c = 1$ (one migration) to those with $c = 2$ (two migrations) using Akaike Information Criterion (AIC) [11]. The model with the lowest AIC value is assumed to be the most parsimonious, and the difference in AIC between the best model and other models ($\Delta$AIC) quantifies how much more parsimonious the best model is.

#### Barren-ground caribou in northern Canada

Caribou are one of the most well-studied species in the animal kingdom [28, 86, 93]. The many subspecies and ecotypes of caribou exhibit different life history and foraging strategies [69], and barren-ground caribou herds in the North American Arctic are notable for their migratory behaviour [30, 38, 60, 89]. Our caribou data were collected for the Qamanirjuaq herd, which ranges across Nunavut’s Kivalliq region for much of the spring and summer. This herd moves annually between their more southern winter grounds and their calving and summer ranges further north. Caribou do not always display high inter-annual fidelity to their wintering grounds [35] but, in part due to the gathering of large herds which facilitates social learning, the herd has displayed high fidelity to their calving grounds for at least 40 years [39]. Pregnant females that arrive on the calving grounds give birth to their calves shortly after, and dramatically reduce their movement for up to two weeks [17, 64]. Identifying the temporal extent of barren-ground caribou migration has management implications, especially as climate change and anthropogenic modifications to the landscape alter the phenology and availability of their food resources [14, 64]. Many efforts have been made to identify these timings in other herds [15, 17, 43, 58, 89].

We fit the MMCP model with $c = 1$ to data describing the spring migrations of barren-ground caribou. Caribou were pursued via helicopter and immobilized via net-gunning, before being fitted with a GPS collar [64]. Following approved protocols, caribou were collared between 2006 and 2016 and in total, we included 35 adult females in the dataset, of which 22 were tracked for more than 1 year. We isolated each individual year and subsetted the data such that any locations after July 1 of that year were omitted. We chose this date because it is after the calving period [64] but earlier than the onset of fall migration [59]. The fix rates of each individual in the dataset varied from 1 h to 1 day, so we rarefied all the data to a 1-day fix rate for consistency. We required estimated migrations ($t_2-t_1$) to be longer than 14 days for all individuals. Similarly to the ferruginous hawk dataset, we removed any paths with significant spatial (150 km between two consecutive recorded locations) or temporal (any 14-day period without recorded locations) gaps from our dataset. Since parturition typically takes place shortly after the end of spring migration for Qamanirjuaq caribou, we compared our estimates for $t_2$ to model-estimated calving dates for each caribou using the method of DeMars et al. [17]. This simple approach uses a broken-stick linear regression to identify multi-day periods when adult caribou stop moving, suggesting they are tending to their offspring. While similarity between our model’s estimates for $t_2$ and calving-based estimates for $t_2$ do not guarantee that our model is accurate, dissimilarity between these two estimates certainly suggest that our model may not always be able to identify migration events from caribou data.

#### Brown bears in northern Canada

Brown bears are opportunistic omnivores with a wide distribution across North America, Europe, and Asia [72]. Brown bears in the Canadian Arctic are unique in comparison to their conspecifics worldwide, exhibiting many adaptations to harsh environmental conditions [23]. Brown bears are not considered migratory, but bears living in the Mackenzie River Delta region of northern Canada display annual home range shifts [25], and some perform temporally oriented navigations to food resources visited a year prior [88]. We used brown bear movement data from the Mackenzie Delta to evaluate if our model would identify any migratory patterns in what biologists view as a non-migratory species. Brown bears were captured, immobilized, and equipped with GPS collars between 2003 and 2006 [25]. These collars were set to record GPS locations at a 4-hour fix rate, and once again, we rarefied all our data to 1-day fix rates for consistency with other case studies. Brown bears in the Canadian Arctic spend up to 6–7 months of the year in a den where they hibernate [45, 65, 70]. In total, we included 25 bears (20 females and 5 males) in our analysis.

Given the broad definitions of migration [19] and the simplicity of our model, we saw value in searching for population-level trends in periods of high-intensity movement within the brown bear dataset. We fit the model with two migratory periods ($c = 2$) to every individual year in the dataset (many individuals had more than one complete year of data), under the assumption that bears would need to exhibit at least two periods of high-intensity movement to complete their theoretical migratory cycle. We evaluated whether the three regions identified as “non-migratory ranges” by the model (i.e., all locations before time $t_1$, all locations between times $t_2$ and $t_3$, and all locations after time $t_4$) were indeed spatially disjoint, as one would expect from “true” migration [6, 19]. We determined the degree of spatial overlap between these ranges by calculating Bhattacharyya’s affinity for each pair of ranges [29]. We calculated Bhattacharyya’s affinity using the overlap function from the ctmm R package [31].

## Results

Results from each migratory path, simulated or real, can be found in Additional file 1, which is available at github.com/pthompson234/migrationmodelling.

### Simulation analysis

All of our models produced parameter estimates for each of the 150 simulated paths without any unsolvable convergence issues. On average, our model (0.150 s) was the second-fastest approach, only trailing the first passage time model (FPT; 0.134 s). The other five models, listed in order of average computational speed, were the nonlinear least squares model for NSD (NLS; 0.344 s), the mechanistic range shift analysis (MRSA; 1.208 s), the behavioral change point analysis (BCPA; 2.216 s), the Bayesian partitioning of Markov models approach (BPMM; 2.368 s), and the Bayesian piecewise NSD regression model (BPWR; 14.86 s).

**Fig. 1:**
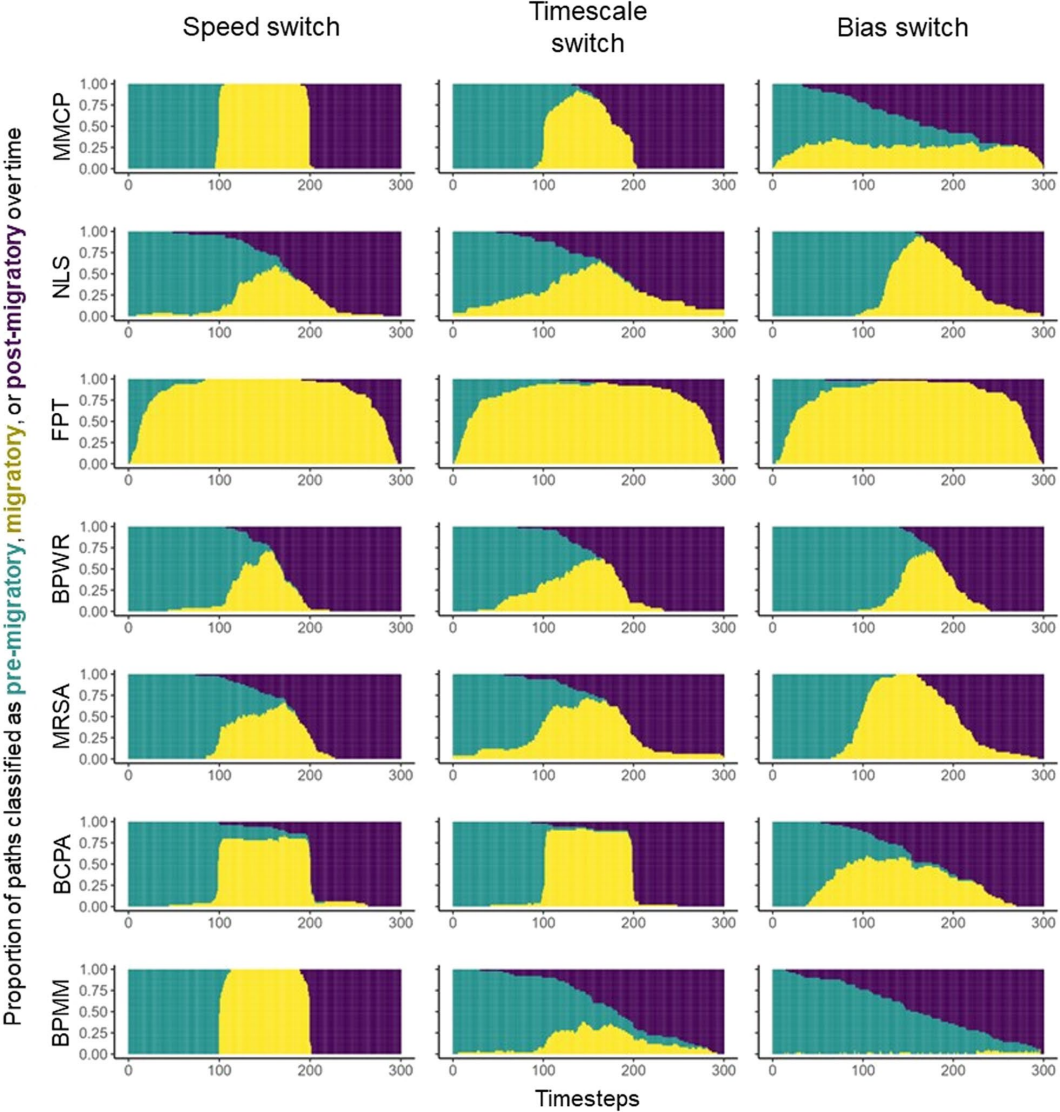
Estimated beginning and end of migration ($\hat{t_1}$ and $\hat{t_2}$ for 150 simulated migratory movements generated according to three different mechanistic models (speed switch, timescale switch, and bias switch), all with “true” migrations lasting from $t = 100$ to $t = 200$. Each panel represents one of the competing migration modelling techniques, organized by rows: MMCP (our multi-metric change-point approach) NLS [10], FPT [58], BPWR [99], MRSA [42], BCPA [40], and BPMM [37]. The y-axis of each panel represents the proportion of paths that were estimated as being migratory along each point of the x-axis (time). Models that effectively identify migration should display three vertical stripes of teal, yellow, and purple, from left to right

Our model estimated $t_1$ and $t_2$ very accurately for some of the simulated migrations but not for others. Specifically, our model was very precise and accurate for simulations generated by the “speed switch” model, where almost every path was perfectly partitioned into migratory and non-migratory bouts (Fig. 1). The BCPA and BPMM approaches were also very effective in this context. Our model was less consistent for the “timescale switch” paths, but was still more accurate than many competing approaches. The BCPA performed very well in this scenario. Our model performed very poorly, appearing almost random, when fit to the “bias switch” paths. The MRSA appeared to be most effective here, although it occasionally overestimated the duration of migration. The NLS and BPWR approaches, which both rely on net squared displacement as a metric, also performed fairly well in this case.

Our model was robust to small amounts of error but failed when spatial error was larger than the average step length (Additional file 1: Figure S1). When we fixed $\sigma _e = 1$, which produced random Gaussian error with variance 1 in the x and y axis, our results were very similar to the case displayed in Fig. 1, where no error was present. When we increased $\sigma _e$ to 25, producing an average error greater than 5 spatial units, model performance was noticeably worse in all conditions (Additional file 1: Figure S2). The MRSA (mechanistic) and BPWR (phenomenological) models were relatively resistant to error within the range we tested, producing similar results for all values of $\sigma _e$ (Additional file 1: Figs. S1, S2).

### Case study: ferruginous hawks

**Fig. 2:**
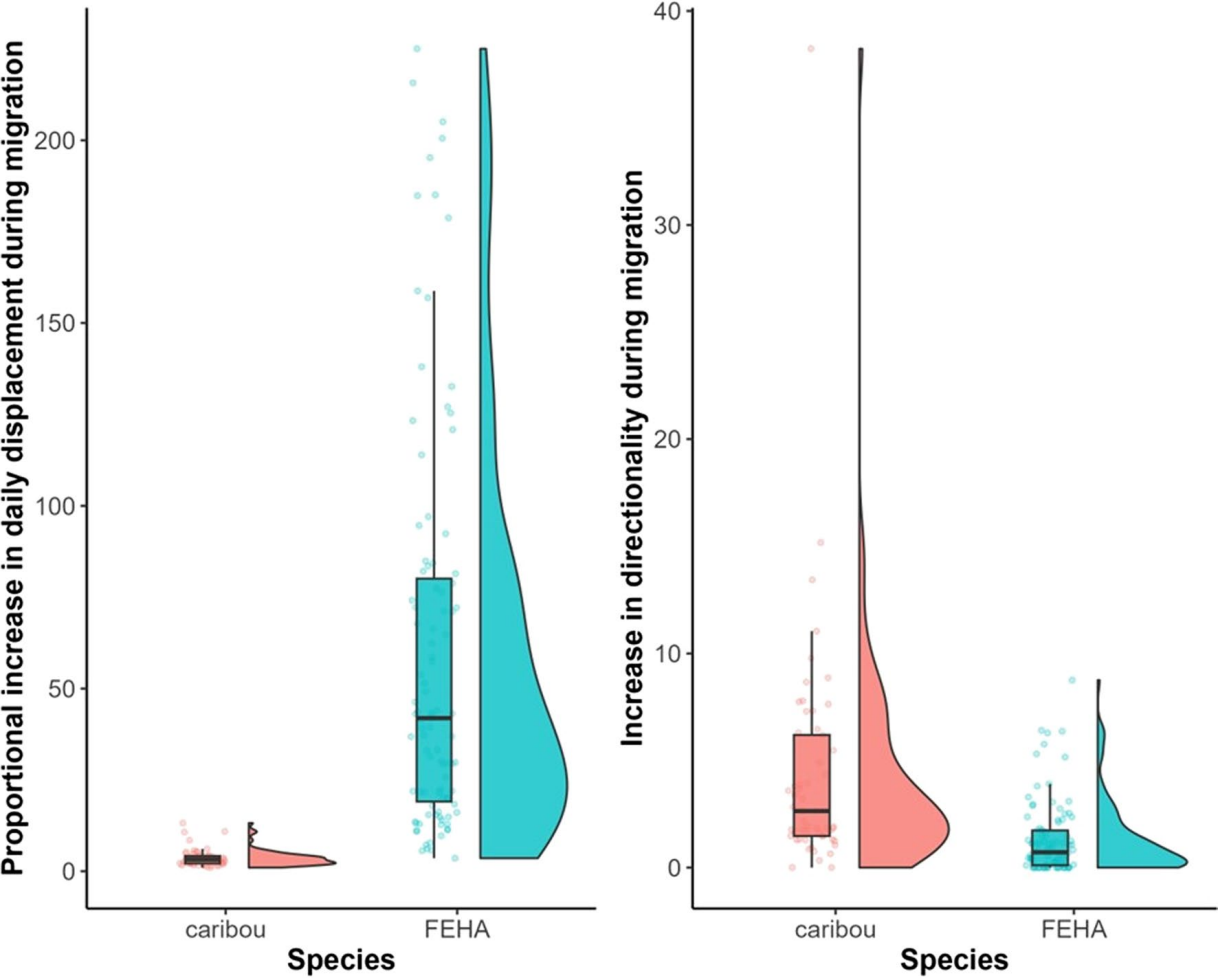
Raincloud plots describing the variation in migratory behavior between and within two of our case studies (caribou, in red, and ferruginous hawks or FEHA in blue). The left panel displays values of $R = \frac{\rho _1 + \rho _0}{\rho _0}$, which measures how much more quickly animals move during migration, and the right panel displays $\kappa _1$, which represents the increase in directional persistence observed during migration. The values for ferruginous hawks were obtained by fitting our model with $c = 1$

We identified 99 unique ferruginous hawk migrations (35 fall, 64 spring). Our model precisely identified the beginning and end of these migratory movements (Additional file 1). Ferruginous hawks rapidly increased their step lengths during migration but did not display as much change in their directionality (Fig. 2). The average value of *R*, which approximates the proportional increase in mean displacement during migration, was approximately 60.14 for ferruginous hawks. Outside of migration, ferruginous hawk step lengths averaged 4.562 km (the mean of all $\rho _0$ estimates for each migration), and this increased by 140.2 km (the mean $\rho _1$ estimate) during migration. The median 95% confidence interval widths for all six of our model parameters (1.525 days, 1.485 days, 1.008 km, 129.2 km, 0.242, and 1.458 for $t_1, t_2, \rho _0, \rho _1, \kappa _0$, and $\kappa _1$, respectively) suggests that all model parameters are usually estimable (Additional file 1). Independent runs of the parametric bootstrapping algorithm produced similar results for the same data.

**Fig. 3:**
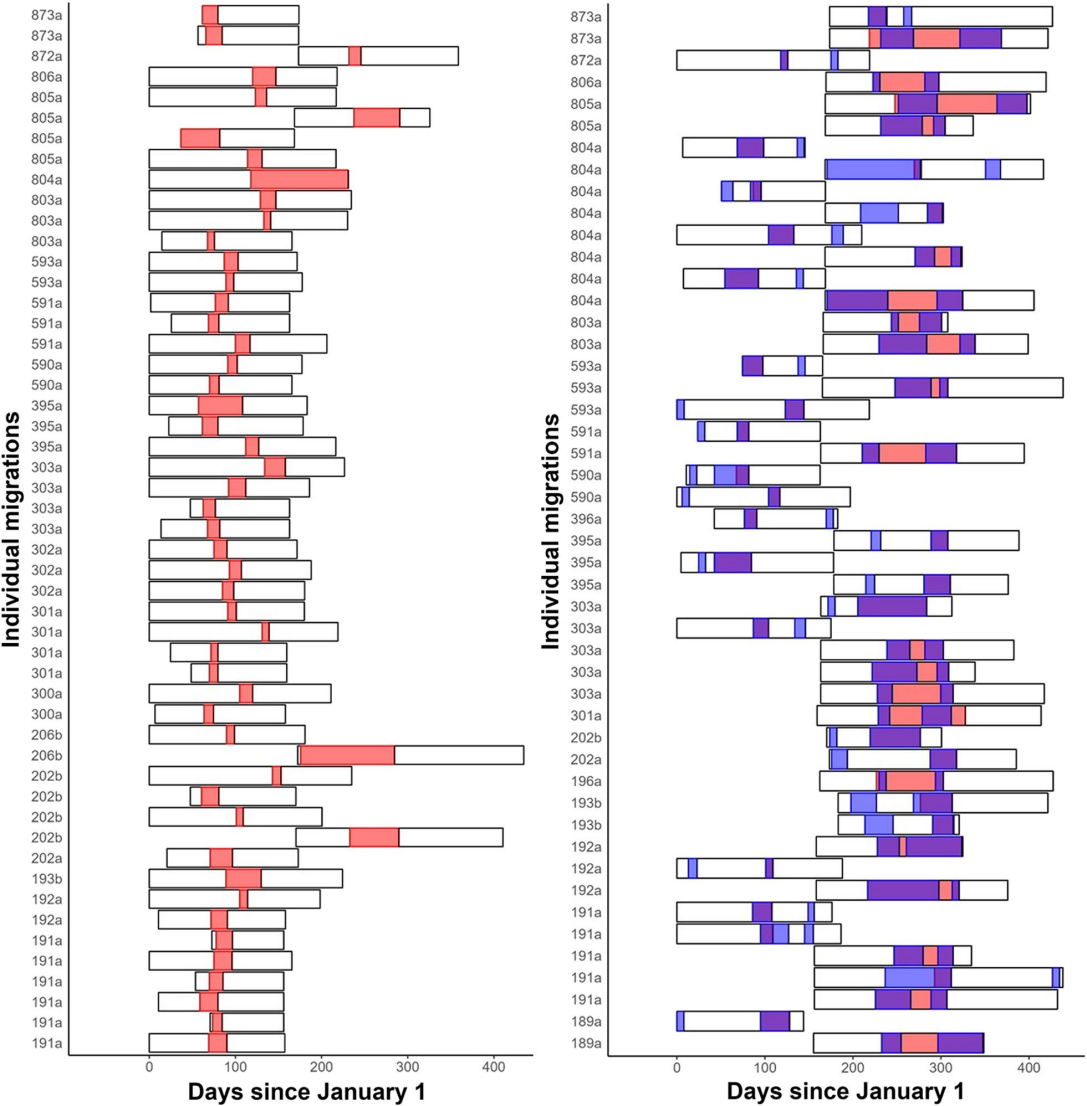
Distribution of migration timings for individual ferruginous hawks in the Great Plains. The panel on the left includes all individual migrations for which Akaike Information Criterion (AIC) favoured the $c = 1$ model (i.e., one uninterrupted migration), whereas the right panel includes all $c = 2$ individuals (i.e., migrations interrupted by stopovers or preceded by pre-migratory dispersal). Black outlines represent the temporal extent of each track. The red and blue (only seen on the right panel) regions of each bar represent the times identified as migration by the $c = 1$ and $c = 2$ models, respectively. Purple regions indicate concordance between the two models

**Fig. 4:**
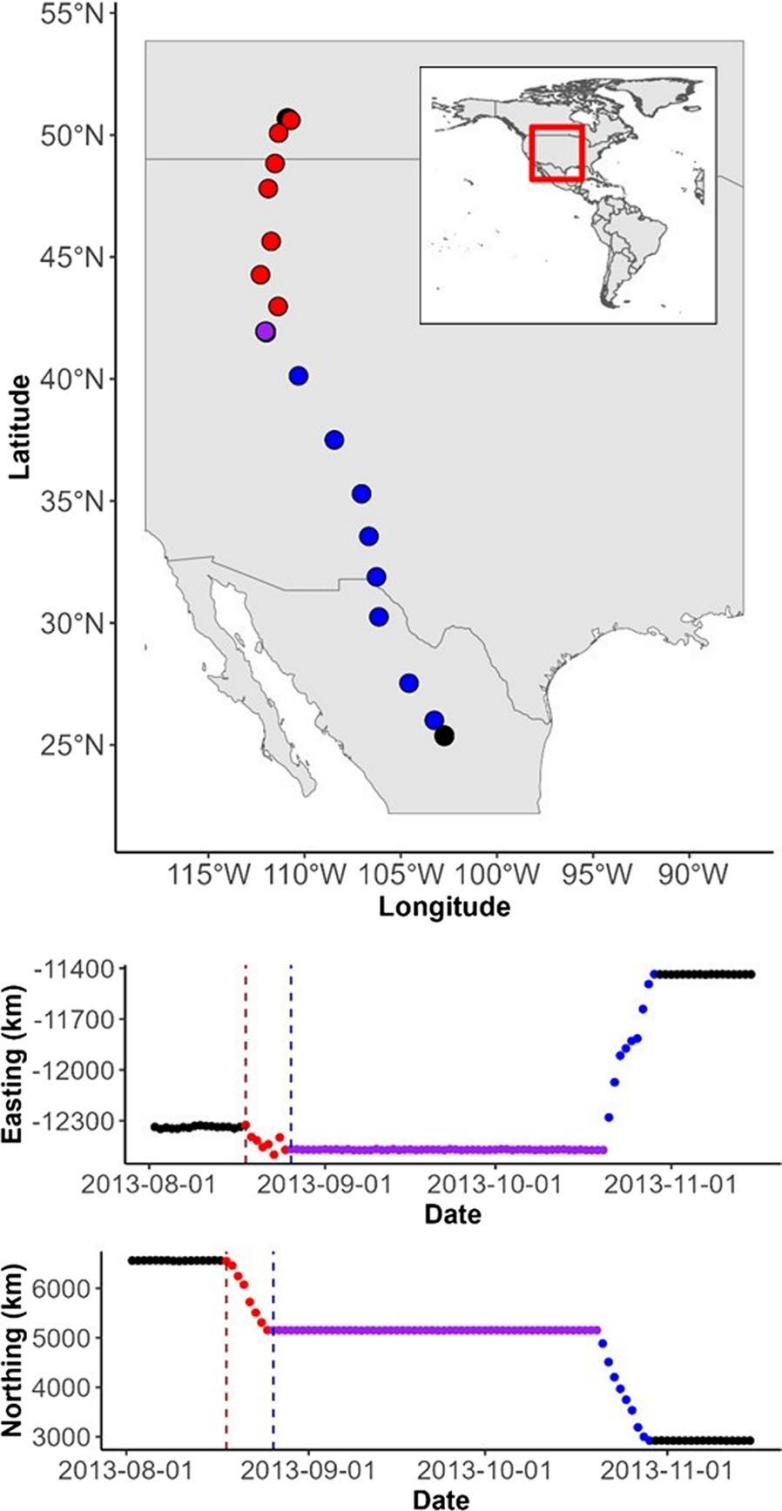
Movement path of a ferruginous hawk (hawk ID 196a; fall 2013) performing a fall migration from its breeding territory in Alberta, Canada, to its wintering grounds in northern Mexico, including a stopover. Locations are plotted on three separate axes (from top to bottom: x–y, t–x, and t–y) and are coloured based on behavioural state as classified by the $c = 2$ model (black = nonmigratory; red = migration, pre-stopover; purple = stopover; blue = migration, post-stopover). Vertical dashed lines on the bottom two panels represent the migration timings estimated by the $c = 1$ model, where the second part of the migration is omitted entirely

The $c = 2$ model identified the timing and location of stopovers and pre-migratory movements in ferruginous hawks (Fig. 3). Fall migrants frequently exhibited stopover behaviour, sometimes migrating for > 1000 km before drastically and temporarily reducing their movement rates (Fig. 4). The $c = 1$ model occasionally identified only one portion of the fall migration in these cases, but sometimes ignored the stopover altogether. The $c = 2$ model was often identified as more parsimonious than the $c = 1$ model (based on AIC) when stopover or pre-migratory behaviours were present (Additional file 1). For example, the migration depicted in Fig. 4 had much lower AIC values with the $c = 2$ model ($\Delta$AIC = 319.9).

### Case study: barren-ground caribou

After filtering the caribou data, we retained 57 individual spring migrations to which we fit the $c = 1$ migration model. Caribou did not increase their daily mean displacement rate as much as ferruginous hawks during migration, as the mean value of *R* was 3.66 (Fig. 2). However, 54 of the 57 migrations displayed significantly higher directional persistence on migration, with 95% CIs for $\kappa _1$ excluding 0. The median confidence interval width for $t_1, t_2, \rho _0, \rho _1, \kappa _0,$ and $\kappa _1$ were 10.05 days, 8.525 days, 1.787 km, 9.555 km, 0.557, and 4.329, respectively.

**Fig. 5:**
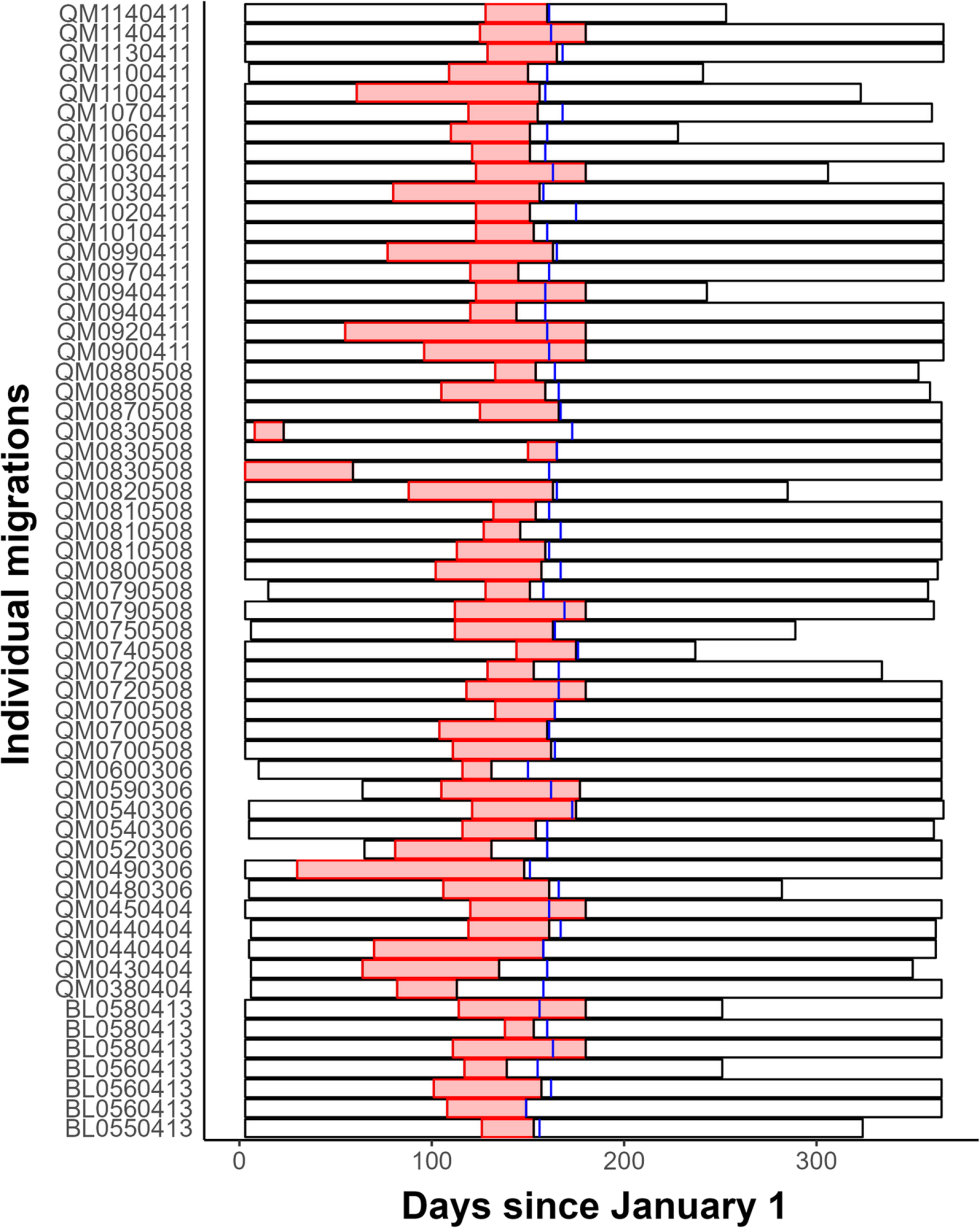
Distribution of migration timings for individual caribou in the Canadian Arctic. Black outlines represent the temporal extent of each track. Red bars represent the migratory period estimated by our model. The blue vertical line in each bar represents the calving date as estimated by the method of DeMars et al. [17]

Our model typically identified biologically reasonable migratory periods from the herd, but for some individuals, our model misidentified a period of sustained movement on the wintering grounds as migration, rather than identifying the spring movement to the calving grounds. In these cases, the estimated parturition date was very different from our model’s estimate of $t_2$ (Fig. 5).

### Case study: brown bears

**Fig. 6:**
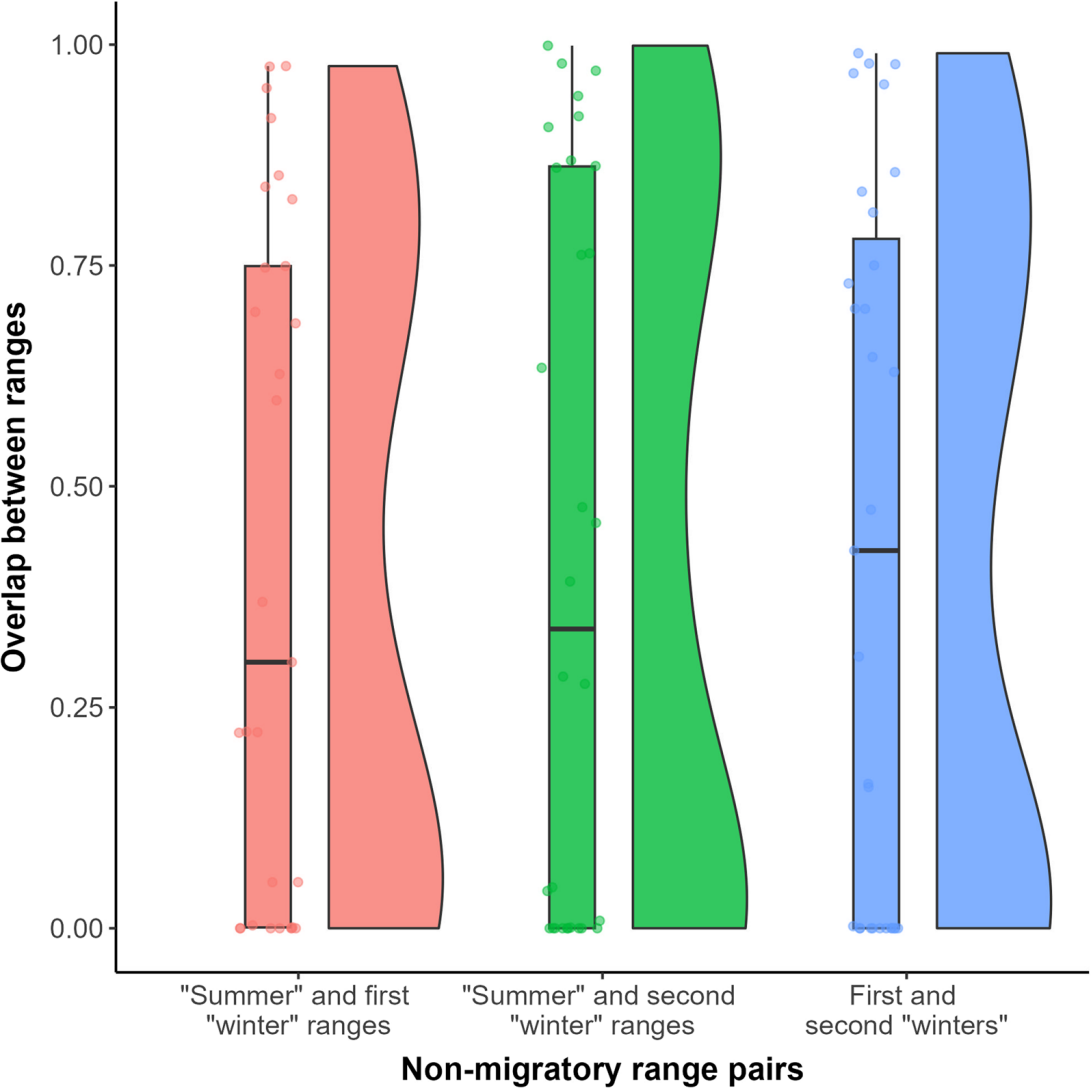
Raincloud plots describing the distribution of spatial overlap (calculated using Bhattacharyya’s affinity) between segments of brown bear movement paths identified by our model as “non-migratory”. For animals that migrate back and forth between two disjoint spatial areas over the course of a year, we would expect almost no overlap between consecutive non-migratory ranges (the red and green plots), and very high overlap between the first and third non-migratory ranges (the blue plot)

We fit the $c = 2$ model to 36 different bear-years and could not identify any trends from the sampled animals. The model identified periods in which brown bears moved more quickly and/or less tortuously for a number of days or weeks but there was no consistency within the population as to when these periods took place or how long they lasted. According to our model, 12 bears “migrated” for over 75% of the active season (Additional file 1). For eight bear-years, the duration of one of the “migratory” periods was 7 days or shorter. Spatial overlap metrics calculated between the three periods of “non-migratory” movement for each bear indicate that many bears did not move between spatially disjoint areas during their “migrations” (Fig. 6). While the pre-migratory and post-migratory ranges, which are expected to be similar in true migrants, displayed higher overlap on average than the other two pairs of ranges, these metrics all varied significantly between individuals. Some bears had three non-migratory ranges that did not overlap at all, while others displayed $>90\%$ overlap between all three non-migratory ranges (Additional file 1).

## Discussion

Animal migration is a complex behavioral process that we can often only observe through the lens of discrete-time location data [53]. Human-induced rapid environmental change may have particularly adverse effects on migratory animals, as migration appears to be on the decline globally [46], highlighting the importance of understanding and characterizing this phenomenon. Ecologists have designed a variety of approaches that can segment animal location data into migratory and non-migratory periods. Here we introduce the multi-metric change-point model and demonstrate situations in which is it useful for achieving this goal. The MMCP model is a simple approach that identifies changes in the observed time series of step lengths and turning angles, two fundamental and well-known movement metrics [68]. The approach is computationally fast and in addition to behaviourally segmenting the animal’s movement path, it estimates parameters that quantify how the animal changes its movements during migration. We also introduced a method for obtaining confidence intervals for these timings, which can be difficult or impossible with other techniques. Our simulation analysis suggests that there are situations where the MMCP model estimates migration timings more accurately than other existing approaches, but there are also situations where it performs very poorly (Fig. 1). While we acknowledge that the model may not be universally applicable to all migratory animals, our results suggest that fitting this model to animal tracking data can generate rigorous and insightful results in some cases.

Unsurprisingly, it appears that the MMCP model is most effective when an animal’s migration involves a marked change in speed or directionality. In particular, the model seems to work very well for migrations that are defined by an increase in movement speed. These two types of migration (increase in speed or increase in directionality) connect to the two definitions of individual migration provided by Dingle and Drake [19], but they are undoubtedly not the only kinds of migration. The “bias switch” model discussed in Gurarie et al. [41] may only be a theoretical simulation, but is proof that migration (as most ecologists would define it) can occur without changes in step lengths or turning angles. Indeed, our model was extremely ineffective at estimating when migration began and ended for “bias switch” simulations (Fig. 1). From a data analysis perspective, data like what was generated by the “bias switch” process could be made more suitable to the MMCP model through subsampling and re-calculating step lengths and turning angles, as they may have different properties at lower fix rates [51, 71, 74], but we acknowledge that throwing out data to make these a priori corrections is inconvenient. In these cases, models that analyze more phenomenological metrics like net squared displacement may be more appropriate. Phenomenological and mechanistic models also appeared to perform better when spatial error was high (Additional file 1: Figure S2). For datasets where spatial location error is considerable, the MMCP model may not be the preferred approach for identifying the temporal extent of migration.

A notable conclusion from our simulation analysis is that none of the models were effective at identifying migration from all three simulation processes. The MMCP model was extremely accurate for the “speed switch” process but not for the “bias switch” process. The same can be said for the BCPA, which was the most effective model for the “timescale switch” process. The MRSA, BPWR, and NLS models were all more effective than the rest at identifying migrations from the “bias switch” process, but were less effective for the other simulations. In practice, we often do not know the underlying behavioural and cognitive mechanisms driving animal migrations. While assessing the width of confidence intervals (or credible intervals, if one takes a Bayesian approach) can be informative about the accuracy of parameter estimates, it can still be difficult to truly know whether a model is right or wrong. To address this difficult problem, we advocate for the use of multiple techniques at once, with agreement between models suggesting an accurate identification of the migratory process. Our model can theoretically be adapted to a Bayesian framework, and we encourage practitioners to incorporate a priori information about their study systems if appropriate, whether it be through frequentist (e.g., bounding parameters during optimization) or Bayesian (e.g., using prior distributions) means. In our caribou case study, we compared estimated $t_2$ values to calving dates estimated by a different technique, which allowed us to identify movement paths that the MMCP model may have failed to estimate.

Our model yielded valuable information about migratory behaviour in ferruginous hawks. The tendency for ferruginous hawks to exhibit long-term stopover behaviour during their fall migrations has been documented in the literature [95, 96]. We expanded on existing knowledge using the MMCP model, which quickly and efficiently divided hawk movement paths into migratory and non-migratory segments. Our results suggest that ferruginous hawks almost always include a long-term stopover in their fall migrations, and almost never do so in their relatively quick spring migrations (Fig. 3). The marked difference between spring and fall migrations likely came about due to the difference in reproductive motivations during each of these seasons. Optimal flight speed theory suggests that birds migrating to their breeding site will move faster than birds migrating away from it, owing to the high competition for breeding territories with conspecifics in the spring [48]. Ferruginous hawks are also known to exhibit pre-migratory behavior where they disperse away from their breeding grounds in a different direction before truly beginning their migrations south [96]. While our model appeared to identify both these movement types with $c = 2$, discerning pre-migratory behavior from long-term stopover behavior is difficult without the additional biological context. Depending on how these behaviors are defined in different systems, some sort of post-hoc analysis may be required to discern them.

The MMCP model quantified exactly how an animals’ movement patterns changed during migration. By combining step lengths and turning angles to identify migration in ferruginous hawks and barren-ground caribou, our model facilitated a connection between parameter estimates and the biological definitions of migration for these species. We used $R = \frac{\rho _1 + \rho _0}{\rho _0}$ and $\kappa _1$ to quantify proportional increases in daily displacement and directionality during migration, respectively, as they can be easily compared between species. Ferruginous hawks moved in a more directed manner during migration, but also moved much more quickly (Fig. 2). Migratory movements carried out over significantly larger scales than observed during range residency correspond to the second migration definition provided by Dingle and Drake [19]. Migratory barren-ground caribou dramatically increased the directional persistence of their movement during migration but did not increase their daily mean displacement as proportionally high as the hawks did (Fig. 2). These migrations resembled the first (undistracted and persistent) definition of migration from Dingle and Drake [19]. It may not be surprising (or novel) that ferruginous hawks, an aerial migrant, move further than terrestrial migrants like caribou during their migrations, but using our model’s parameters and their derived quantities (e.g., *R*) could facilitate comparison across different levels (e.g., different populations of ferruginous hawks, or different demographic classes within the same population, when these data are available) that expand our understanding of how these animals move.

Our model identified periods of increased daily displacement and/or directional autocorrelation from the brown bear data, but it is unclear whether these movements represent migration. Some of these bears did not even leave their original “pre-migratory” ranges during their “migrations”, as suggested by the prevalence of high spatial overlap values between all three non-migratory ranges (Fig. 6). In general, these overlap values were highly variable between all pairs of non-migratory ranges, and it was difficult to discern any pattern at all. This may simply be a consequence of our model’s inability to identify biologically meaningful behavioural periods from the data, but the variability in overlap values is not surprising for this species. Brown bears are highly individualized animals that display individual variation in their diets [24, 79], movements [16, 73], interactions with humans [7, 8], and more. While we believe it is hard to conclude from our analysis that brown bears are migratory, individual variation is likely to affect migratory patterns in many ways, and although we did not have sufficient demographic data to analyze these trends in any of our case studies, we hope that these variables are considered whenever analyzing migration on the individual scale.

An essential part of our model is the change-point algorithm used to identify the values of $t_1$ and $t_2$ that maximized our likelihood function. Determining migration from a time-series of step lengths and/or turning angles is certainly not unique but the method we used to identify these optimal values is different from all existing change-point algorithms. While it is not mathematically exact, it is more flexible than many similar approaches in its ability to identify path segments with nonindependent metric distributions. Our algorithm is much more efficient than an exhaustive search, which would be computationally unfeasible for many datasets. Change-point identification has applications in a wide range of scientific fields [90], and we suggest that our algorithm could be applied to a diversity of time-series analysis problems, not just migration.

Our model achieved the sought-after goal of determining when animals begin and end their migrations. By parameterizing time-dependent step length and turning angle distributions, we generated results that are easy to interpret biologically. Migration incurs an elevated risk to the negative effects of anthropogenic global change ([98]; but also see [100]). Specifically, many animals are arriving at their breeding grounds earlier to capitalize on global warming-induced advances in green-up and prey availability [44, 64]. Many ecologists expect (or are already observing) changes in when, where, and how animals migrate [91, 98]. The MMCP model provides unbiased, quantitative information on all three of these characteristics.

### Supplementary Information

**Additional file 1.** Results from simulation and empirical analyses, including parameter estimates, confidence intervals (where appropriate), and negative log-likelihood values for each individual path included in our analyses.

## Data Availability

All code used to perform the analyses in this article (as well as samples of data from each case study to which the code can be applied) are included in the following Github repository: https://github.com/pthompson234/migrationmodelling. The repository also contains a short vignette document that demonstrates our workflow and can hopefully be adapted by practitioners. Sample animal data have had all locations adjusted. This does not affect the analysis as all values have been shifted by the same amount.
