# Supplementary material for: Simultaneous estimation of the temporal and spatial extent of animal migration using step lengths and turning angles
Source: Mov Ecol. 2024 Jan 8;12:1. doi: 10.1186/s40462-023-00444-8 (PMC10775566; doi:10.1186/s40462-023-00444-8)
Supplement: Supplementary file 1 — Additional file 1. Results from simulation and empirical analyses, including parameter estimates, confidence intervals (where appropriate), and negative log-likelihood values for each individual path included in our analyses. [file 40462_2023_444_MOESM1_ESM.pdf]

Appendix: Simulation analyses

Model comparison analysis

In addition to fitting our MMCP model to all of the simulated paths, we also used six competing approaches to estimate t_1 and t_2. The model designed by Bunnefeld et al. (2011) uses nonlinear least squares (NLS) to fit a curve to the net squared displacement (NSD) profile of a movement path over time. Bunnefeld et al. (2011) model NSD as a function of time using three paramters: the intensity of migration δ, the temporal "centre" of migration θ, and the "quarter-duration" of migration ψ. The model takes a logistic form:

$$\text{NSD} = \frac{\delta}{1 + \exp \frac{\theta - t}{\psi}}. \tag{3}$$

We fit the model using the `nls` function from the R software. We calculated the beginning and end of migration using $\theta - 2\psi$ and $\theta + 2\psi$, respectively (Bunnefeld et al., 2011). If the optimization process did not converge, we changed the initial parameter guess for ψ from 10 (our default value) to 1, which always seemed to resolved the problem.

First passage time (FPT), which measures the amount of time required for an animal to travel a certain distance, has been used to identify changes in movement behaviour on many scales (Johnson et al., 1992; Fauchald and Tveraa, 2003; Le Corre et al., 2014). This distance must be user-defined beforehand, requiring unique assumptions for every dataset (Barraquand and Benhamou, 2008). We employed the approach from Le Corre et al. (2014), who used the penalized contrast algorithm (Lavielle, 2005) to identify change-points in first passage time (FPT) values over time. We calculated FPT at any time t as the amount of time required for the animal's NSD to exceed some radial threshold D. We set D by maximizing the variance of $\log$ FPT for a sequence of candidate radii

(Fauchald and Tveraa, 2003). We selected our optimal D for each simulation separately, choosing among multiples of 5 km increasing to 100 km (5, 10, ..., 95, 100). Once we picked D and calculated the FPT time series for each simulation, we applied the penalized contrast method to identify the optimal FPT change-points (Lavielle, 2005). We used the `lavielle` R function from the `adehabitatLT` package to run the penalized contrast algorithm (Calenge, 2006), following the methods of Le Corre et al. (2014), with the exception being that we fixed the number of change-points to 2.

We applied the Bayesian piecewise regression method employed by Wolfson et al. (2022) to our simulated paths. This model identifies change-points in an animal's NSD over time by identifying segments of the data in which the mean NSD is distinct from other segments. The model required us to identify the desired number of segments we wished to identify, which will always be equal to $2c + 1$. We only fit the $c = 1$ model to simulated paths and chose to identify three separate segments as a result. We used the `mcp` ("multiple change point") function from the `mcp` package to fit the models, which uses Bayesian Markov Chain Monte Carlo (MCMC) sampling to estimate the posterior distribution of the change-points (Lindeløv, 2020). We derived $\hat{t_1}$ and $\hat{t_2}$ as the posterior median for each change-point.

We fit the mechanistic range shift analysis (MRSA) model, designed by Gurarie et al. (2017), to our simulated paths using the R `marcher` package. We fit the model to simulated movement data using the `estimate_shift` function from the `marcher` package. The function directly estimates the beginning and duration of the range-shifting event using a likelihood-based optimization technique, so we simply added these two parameters together to calculate $\hat{t_2}$).

We used behavioral change point analysis (BCPA; Gurarie et al., 2009) to identify change-points in the "persistence velocity" (step length * cos(turning

angle)) of the simulated movement paths. This entailed using a window sweep algorithm to optimally identify change-points in this metric. This algorithm may identify any number of change-points (usually greater than two, which was our desired number), but using the `ChangePointSummary` function from the `bcpa` package, we were able to identify which change-points were most frequently selected and thus may be the most significant. We identified the two most frequently selected changepoints, ordered them, and reported these values as $\hat{t_1}$ and $\hat{t_2}$.

We also employed a model based on Bayesian partitioning of Markov models (BPMM), a classification-style approach, to our data (Guéguen, 2009; Gurarie et al., 2016). The underlying functions used to run this analyses are included in the `adehabitatLT` R package (Calenge, 2006), but the `waddle` library (Gurarie et al., 2016) includes some additional wrappers that make running the code more convenient. By default, these functions apply model selection to identify the optimal number of partitions, but we only need to find the change-points that partition the data into three segments. We fit the models to the data using the `modpartltraj` function from the `adehabitatLT` package, and then used the `partmod.ltraj` function to derive change-points.

Spatial error analysis

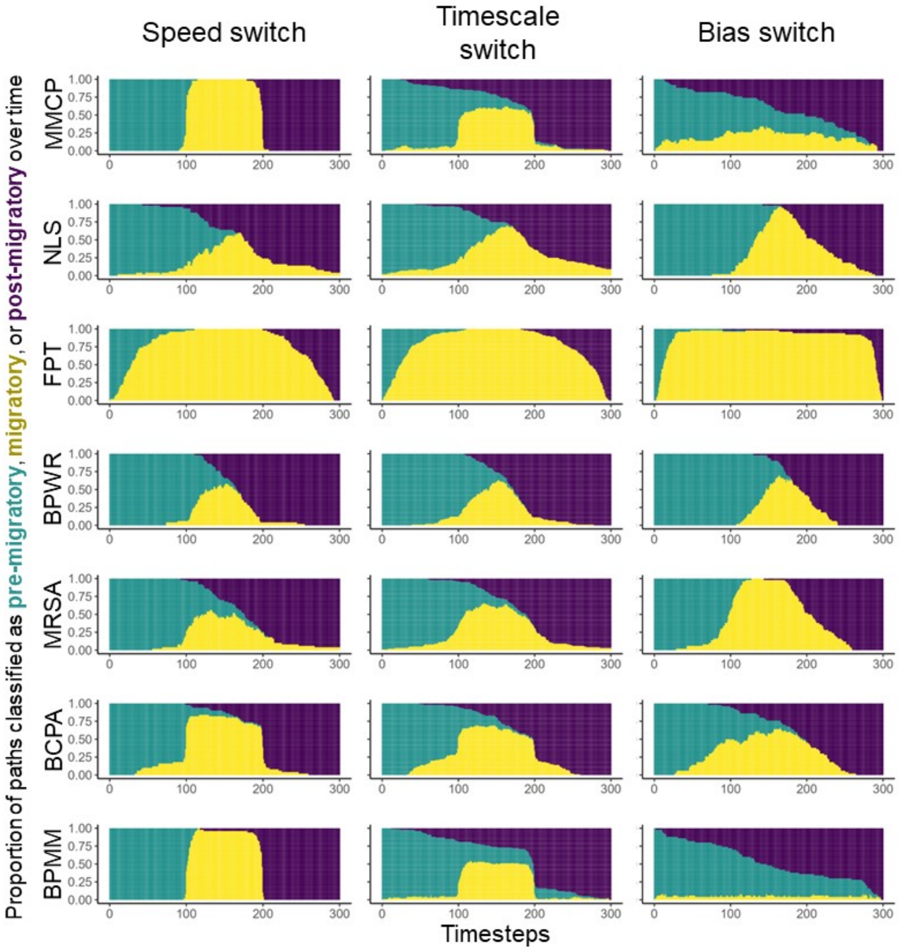

Figure S1: Estimated beginning and end of migration ($\hat{t_1}$ and $\hat{t_2}$ for 150 simulated migratory movements generated according to three different mechanistic models (speed switch, timescale switch, and bias switch), all with "true" migrations lasting from $t = 100$ to $t = 200$ and with artificial location error ($\sigma_e = 1$). Each panel represents one of the competing migration modelling techniques, organized by rows: MMCP (our multi-metric change-point approach) NLS (Bunnefeld et al., 2011), FPT (Le Corre et al., 2014), BPWR (Wolfson et al., 2022), MRSA (Gurarie et al., 2017), BCPA (Gurarie et al., 2009), and BPMM (Guéguen, 2009). The y-axis of each panel represents the proportion of paths that were estimated as being migratory along each point of the x-axis (time). Models that effectively identify migration should display three vertical stripes of teal, yellow, and purple, from left to right.

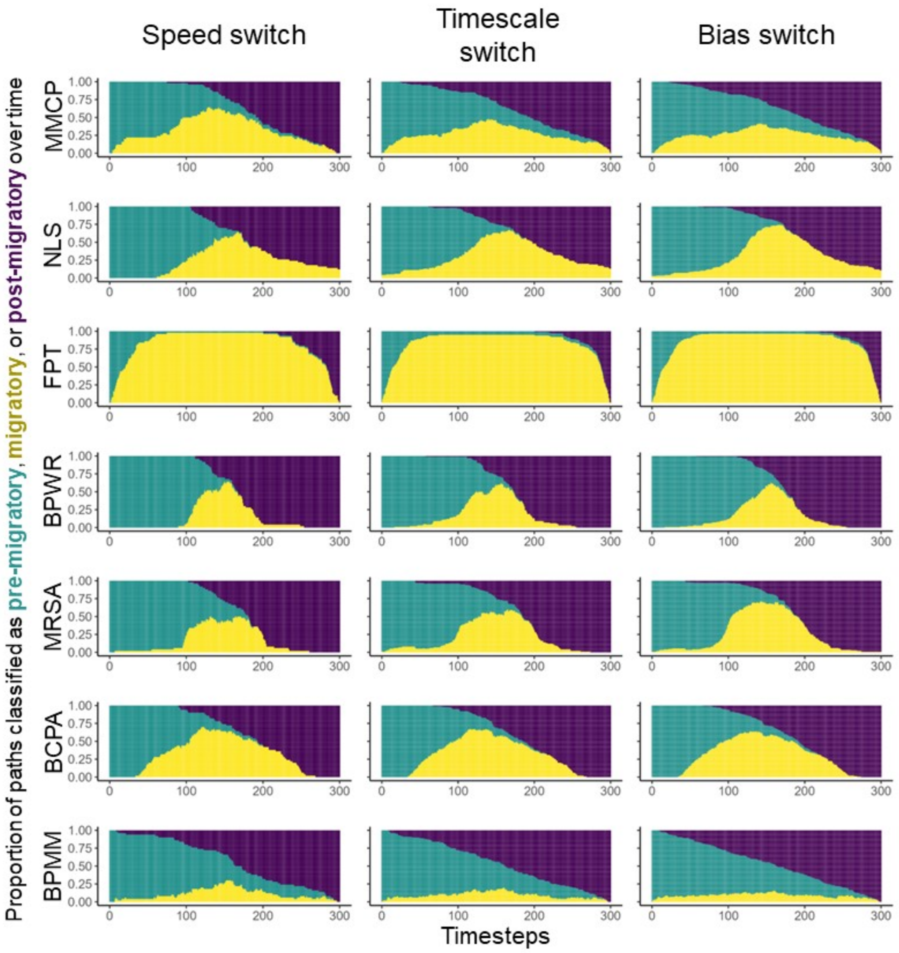

Figure S2: Estimated beginning and end of migration ($\hat{t_1}$ and $\hat{t_2}$ for 150 simulated migratory movements generated according to three different mechanistic models (speed switch, timescale switch, and bias switch), all with "true" migrations lasting from $t = 100$ to $t = 200$ and with artificial location error ($\sigma_e = 25$). Each panel represents one of the competing migration modelling techniques, organized by rows: MMCP (our multi-metric change-point approach) NLS (Bunnefeld et al., 2011), FPT (Le Corre et al., 2014), BPWR (Wolfson et al., 2022), MRSA (Gurarie et al., 2017), BCPA (Gurarie et al., 2009), and BPMM (Guéguen, 2009). The y-axis of each panel represents the proportion of paths that were estimated as being migratory along each point of the x-axis (time). Models that effectively identify migration should display three vertical stripes of teal, yellow, and purple, from left to right.
